# Behavioral Intervention Functions in Type 2 Diabetes Apps: Literature Review

**DOI:** 10.1177/19322968241305646

**Published:** 2024-12-23

**Authors:** Elia Gabarron, Pietro Randine, Eirik Årsand

**Affiliations:** 1 Department of Education, ICT and Learning, Østfold University College, Halden, Norway; 2 Department of Computer Science, Faculty of Science and Technology, UiT The Arctic University of Norway, Tromsø, Norway

**Keywords:** behavioral intervention, health behavior, mobile applications, smartphone, type 2 diabetes

## Abstract

**Background:**

As type 2 diabetes (T2D) is expected to increase, self-management becomes more crucial. Mobile apps are increasingly supporting self-management with tasks like blood glucose monitoring and medication management. Understanding the behavioral intervention functions used by diabetes apps today, is essential for improving future apps and systems for diabetes management.

**Objective:**

To analyze the behavioral intervention functions used in apps for managing T2D that integrate the three main elements: medication management, nutrition tracking, and blood glucose management.

**Methods:**

We conducted a literature review on T2D diabetes apps using SCOPUS, PubMed, and PsycINFO. After screening and removing duplicates, we analyzed app details and behavioral intervention functions based on the Behavior Change Wheel (BCW) framework.

**Results:**

We reviewed 644 scientific publications describing diabetes apps in clinical studies, narrowing it down to 20 studies, including 16 unique apps, after screening and exclusions. These studies were published between 2016 and 2024. Among the identified apps, automatic processing of medication data was reported in one study, while blood glucose data were automatically processed in 13 studies. Nutrition data processing varied. Most apps used *Enablement* and *Persuasion* as behavioral intervention functions, with *Education* and *Training* reported less frequently. *Environmental Restructuring, Incentivization, Coercion, Restriction*, and *Modeling* were not reported as being used in any studies.

**Conclusions:**

This review shows that while *Enablement* and *Persuasion* are common, other behavioral intervention functions seem to be underused or underreported. Future research could explore the potential of integrating additional behavioral intervention functions to enhance diabetes app efficacy and users’ self-management.

## Introduction

Type 2 diabetes (T2D) is a growing global health concern, currently affecting over 400 million individuals worldwide.^1^ Its prevalence is increasing at an alarming rate, with this dramatic rise estimated to impact over 1.27 billion people worldwide by 2050.^2^ Modifiable lifestyle factors such as overweight, diet, and physical inactivity contribute to the development of T2D.^1,3^ Encouraging self-management is essential for individuals with T2D, as it empowers them to manage their health better and improve outcomes.

T2D self-management requires daily tasks, such as monitoring blood glucose levels, managing medication, and adjusting diet.^4^ Mobile phone-based applications or “apps” are increasingly being used to support these tasks, and can enhance self-management of diabetes by integrating various intervention components.^5^

The Behavior Change Wheel (BCW) framework^6^ helps identify strategies that can encourage self-management. The BCW framework comprises nine intervention functions designed to facilitate behavior change.^6^ These functions are *Education* (eg, providing information about the importance of monitoring blood glucose levels); *Persuasion* (eg, using reminders and motivational messages to encourage medication adherence); *Incentivization* (eg, offering rewards for tracking nutrition data); *Coercion* (eg, implementing penalties or reminders for missed medication doses); *Training* (eg, teaching users how to use diabetes apps effectively for self-management); *Restriction* (eg, limiting access to unhealthy foods by promoting healthier alternatives); *Environmental Restructuring* (eg, making glucose-monitoring devices readily available and easy to use); *Modeling* (eg, showing success stories of other patients managing their diabetes effectively); and *Enablement* (eg, providing tools and resources that facilitate self-management, such as easy-to-use apps). The BCW framework has been studied as a basis for improving non-technology-based intervention programs in diabetes,^7-11^ and for creating technology-based interventions as well, such as a phone-based program for women with gestational diabetes^7^; an app to increase physical activity^12^; a virtual assistant for improving adherence to antidiabetic medication in older adults^13^; to create theory-based SMS aimed at reducing the T2D risk,^14^ and to influence food literacy.^15^

Most diabetes apps are complex interventions, integrating multiple components, functions, and strategies aimed at managing various aspects of diabetes and, therefore, improving self-management. While their functions may rely on different theoretical models, clearly reporting of all incorporated functions and their impact, will help in understanding which ones are most effective. To our knowledge, no publications exist analyzing the behavior change functions that are used in apps designed for managing T2D. The objective of this literature review is to explore the use of behavioral intervention functions in a representative sample of publications about apps for managing T2D. The review focuses on apps that integrate one or more of the following three essential management functions: medication, nutrition, and blood glucose management.

## Methods

### Search Strategy

To capture a representative sample of research related to diabetes apps, we conducted a literature review. The search was carried out on February 29, 2024, and covered three databases: SCOPUS, PubMed, and PsycINFO. We limited the search to publications that specifically included the terms “diabetes” and “app” in their title. No year or language limitations were used for this search. The full search strategy is presented in Appendix A.

### Eligibility and Selection Process

All identified references were uploaded to EndNote 20.6 (Clarivate) and Rayyan.^16^ After removing duplicates, a reviewer (EG) conducted the initial screening by reading titles and abstracts. During a second screening, the eligibility of the selected articles was reconsidered and discussed by two reviewers (EG and EÅ) after reading the full text. The inclusion and exclusion criteria are presented in Table 1.

**Table 1.** Inclusion and Exclusion Criteria.

Inclusion criteria	Exclusion criteria
• The article is a primary study that describes an app for T2D management • The described app for T2D has already been developed • The diabetes app covers at least the following three functions: management of medications; nutrition; and blood glucose management	• The article is not a primary study (eg, reviews, editorials, study protocols, etc.) • The article describes an app that is not specifically for T2D management, or that has not been developed yet • The diabetes app does not cover all three functions (ie, management of medication, nutrition, and blood glucose management)

### Data Items and Data Extraction

Two authors (PR and EÅ) extracted the following technical data: app name, operating system of the mobile phone, and type of data collected in four main categories: medication, blood glucose, nutrition, and others (eg, physical activity and blood pressure). Another author (EG) coded the behavioral intervention functions of the apps reported in the included articles according to the BCW framework.^2^

## Results

### Study Selection

**Figure 1.**
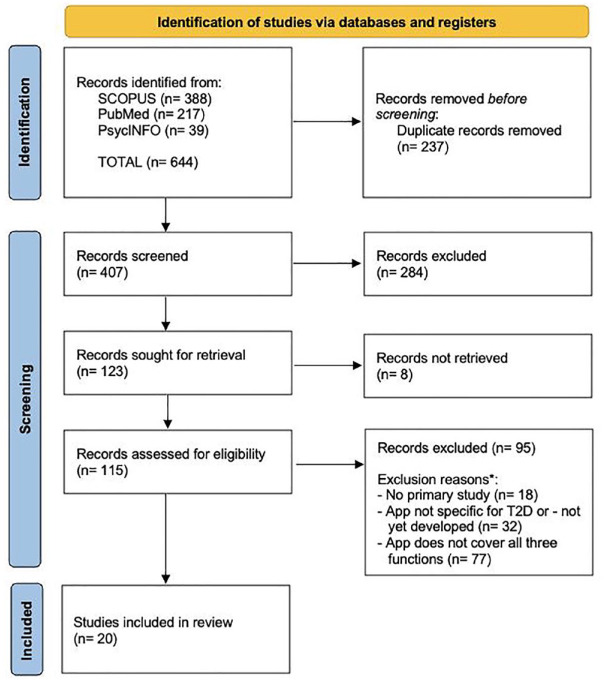
Flowchart diagram of the selection process.

We initially identified 644 articles through the database search. After removing 237 duplicates, 407 articles remained for title and abstract screening. After excluding irrelevant and missing articles, the full texts of 115 articles were reviewed, and 95 articles were further excluded based on the eligibility criteria. The list of articles rejected during the full-text review, along with the reasons for their rejection, is provided in Appendix B. A total of 20 articles were included in this review.^17-36^ (See Figure 1.)

### Main Functions Reported in the Apps in the Included Studies

The 20 selected articles, published between 2016 and 2024, report on 16 unique diabetes apps (see Table 2).

**Table 2.** Summary of the Included Articles (n = 20).

Reference	App name / Operating system	Data processing				Behavioral intervention functions
		Medication	Blood glucose	Nutrition	Other functions	
Kumar et al^31^	One Drop Mobile/iOS	✓ (Via HealthKit)	✓ (Via HealthKit)	✓ (Via HealthKit)	Physical Activity (via HealthKit)	Enablement, Education, Persuasion
Desveaux et al^23^	WellDoc Bluestar/Web-based	✓	✓	✓	Activity levels	Persuasion
Baptista et al^19^	MDC/N.R.	✓	✓	✓ (Healthy eating)	Physical activity Foot care	Education, Persuasion
Steinert A et al^34^	My-Therapy/Android	✓	✓	✓ (Reminders of nutrition)	Physical activity Body weight Blood pressure Reminders	Persuasion
Fisher et al^24^	CONTOUR DIABETES/Both	✓	✓ (automatic transfer from CONTOUR NEXT ONE glucose meter)	✓ (Carbohydrate consumption)	Exercise	Enablement
Modave et al^33^	DiaFit/iOS	✓ (Including reminders)	✓ (Automatic through the wireless smart glucose-monitoring system from Apple Health)	✓ (Carbohydrates, proteins and fats)	Physical activity through synchronization with Apple and Fitbit devices	Enablement, Persuasion
Islam et al^27^	DiaHealth/N.R.	✓	✓ (Automatic from selected Bluetooth enabled devices)	✓ (Including calorie intake and calorie burn)	Blood pressure Weight and height BMI calculation feature	Enablement, Persuasion
Coleone et al^22^	Diabetics Control/N.R	✓	✓ (Using Accu-Check Active glucometer)	✓ (Based on Questionnaire)	Demographic and economic characteristics	Enablement
Xu et al^35^	No name reported (register number: 2018SR446465)/Both	✓	✓	✓	Patient’s symptoms Physical activity Mental status	Training Education Persuasion
Zhang et al^36^	Welltang/N.R.	✓	✓	✓	Exercise Body weight	Training Enablement Education
Handa et al^26^	Smart e-SMBG/Both	✓	✓ (Automatic transfer from several glucometers)	✓	Blood pressure Weight Step count	Enablement
Adu et al^17^	My Care Hub/Android	✓	✓	✓ (Carbohydrate and calorie content)	Physical activities	Enablement, Education, Persuasion
Jeon and Park^28^	DSC/Android	✓	✓	✓	Blood pressure Weight Exercise	Enablement, Education, Persuasion
Kitazawa et al^30^	Health2Sync/Both	✓	✓ (Automatic transfer from the CGM system “FreeStyle Libre Link”)	✓	Physical activity (through a fitness tracking device) Body weight Blood pressure	Enablement, Persuasion
Gong et al^25^	MDC/Both	✓	✓ (From meter with app)	✓ (Healthy eating)	Physical activity Foot care	Enablement, Education Persuasion
Alexiadis A et al^18^	forDiabetes/Both	✓	✓ (Automatic transfer from glucometers Contour Next ONE, Contour Plus ONE, GlucoMen areo, and Beurer GL50)	✓	HbA1c Blood pressure	Enablement
Li et al^32^	IMTOP/N.R.	✓	✓	✓ (Diet behavior, water intake)	Daily exercise behavior	Enablement
Chang et al^21^	Health2Sync/N.R.	✓	✓	✓	Daily behaviors	N.R.
Katz et al^29^	OneTouch Reveal/Both	✓ (Via message)	✓ (Automatic transfer from the OT Verio Flex glucose meter)	✓	N.R.	Enablement, Persuasion
Baptista et al^20^	MDC/Both	✓	✓ (Automatic transfer from the OT Verio Flex glucose meter)	✓ (Healthy eating)	Physical activity Foot care	Enablement, Education Persuasion

Abbreviations: N.R., not reported.

Regarding the three main functions in the apps of our interest (management of medications; nutrition; and blood glucose management), automatic processing of medication-related data is reported by only one of the articles,^31^ and manual data entry is reported in another one.^34^ In the rest of the included articles, no details are provided regarding how the medication-related data were processed. Blood glucose data were processed automatically in 13 articles,^18-22,24-26,28-31,33^ with only one study explicitly mentioning the use of a continuous glucose monitoring (CGM) device, specifically the FreeStyle Libre,^30^ while the rest appeared to focus on Self-Monitoring of Blood Glucose (SMBG) devices. One article reported manual processing of blood glucose.^34^ The processing of nutrition data and reminders of nutrition was explicitly reported to be done manually in four articles.^22,31,33,34^ In the rest of the articles, the processing of nutrition data is reported, but the specific methods are not provided.

Other functions included by these apps involve the processing of physical activity or exercise data^17,19,20,23-26,28,30-32,35^; blood pressure^18,26-28,30,34^; body mass index (BMI), weight, and/or height^26-28,30,34^; foot care^19,20,25^; demographic and economic data^22^; and other symptoms.^35^

#### Behavioral intervention functions included in the apps

All articles reported the use of one or several behavioral intervention functions in their apps (see Table 2), except for one article.^21^

The most commonly reported behavioral intervention function was *Enablement*. This function was reported in 15 of the 20 included articles.^17,18,20,22,24-33,36^ *Enablement* is achieved through techniques such as allowing users to view or monitor their data, receive feedback on their progress, and provide graphical breakdowns of macronutrients consumed by the user. The next most commonly used behavioral intervention function was *Persuasion*, reported in 13 articles.^17,19,20,23,25,27-31,33-35^ Examples of how *Persuasion* is integrated into these apps include the use of prompts and tailored messages, delivering content on an as-needed basis, providing motivational support and encouragement, or offering personalized advice. The integration of *Education* in the apps was reported in eight of the 21 included studies.^17,19,20,25,28,31,35,36^ This intervention function was integrated through the inclusion of educational programs or modules, or by delivering diabetes-related knowledge. *Training* was reported as an intervention function used in two articles.^35,36^ The articles indicate that *Training* was delivered by providing a variety of diabetes self-management strategies and promoting learning skills through the app. None of the 20 included articles have explicitly reported the use of *Environmental Restructuring, Incentivization, Coercion, Restriction*, or *Modeling* as behavioral intervention functions in their diabetes apps.

A visual example of how *Enablement, Persuasion*, and *Education* were used in the apps is shown in Appendix C. The articles reporting the use of *Training* did not include screenshots of the apps and the apps themselves were not publicly accessible; therefore, we are unable to provide visual examples of how *Training* was implemented in these two apps.

## Discussion

### Summary of Findings

**Figure 2.**
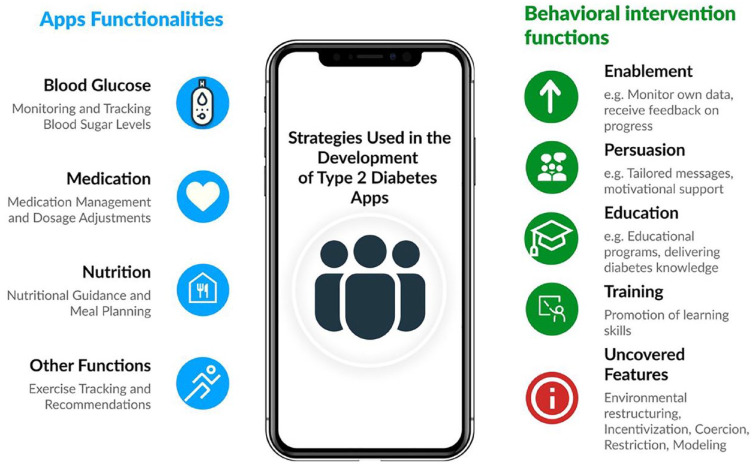
Apps functionalities and behavioral intervention functions in T2D apps.

We have identified 20 scientific articles reporting on 16 unique apps for T2D self-management that integrate medication management, nutrition tracking, and blood glucose management. The intervention functions described in these apps for addressing behavior change include *Enablement, Persuasion, Education*, and *Training*, are aiming to support users in managing their T2D, as graphically summarized in Figure 2. The apps allow users to manually record their medication and food intake, often through questionnaires or built-in features. In 13 studies, glucose data were automatically collected via a connected glucometer, with one explicitly mentioning the use of CGM. Many apps offer additional features like tracking physical activity, HbA1c levels, blood pressure, foot care, and weight.

### Implemented and Underutilized BCW Strategies in T2D Apps

The integration of BCW intervention functions in apps designed for T2D management is key for empowering individuals to adopt and maintain desired health behaviors.^6^ Our review found that the most commonly reported BCW function integrated into T2D apps was *Enablement*.^17,18,20,22,24-33,36^ *Enablement* enhances users’ capability, and it can also be seen as a way to create opportunities for behavior change by providing resources or reducing barriers. In the T2D apps, this intervention function is used by incorporating techniques such as enabling users to track and monitor their own data, which has been found to be a key factor influencing engagement with health apps^37^ and a feature recommended to be included in the development of health apps.^38^ The fact that *Enablement* was the most reported BCW function in T2D apps suggests that these interventions focus on empowering users by providing the tools and support needed for better managing their condition. A restricting issue arises from the need for manual recording method of data, which relies on direct user inputs rather than an automatic process. Of 20 studies, including blood glucose monitoring, 13^18-22,24-26,28-31,33^ reported an automated method for recording data directly from glucometers. The main limitation is reliance on external sources for diet information and the inability to record medication automatically. This has become possible through certain insulin pens with wireless communication.

We identified *Persuasion* as the second most commonly used BCW intervention function in T2D apps,^17,19,20,23,25,27-31,33-35^ to actively influencing and enhancing users’ motivation to adopt desired behaviors. The articles described that *Persuasion* was integrated into the apps by including the use of prompts and tailored reminders or messages. These apps could then deliver content on an as-needed basis, providing motivational support and encouragement, offering personalized advice. These strategies may help reinforce positive behavior change by keeping users engaged and motivated, and promote long-term adherence to healthy habits.^37,38^

The *Education* function was reported as being used by almost a third of the articles.^17,19,20,25,28,31,35,36^ Some health app users as a feature to build knowledge and understand and manage their behavior better.^37^ Therefore, by incorporating *Education*, these T2D apps can increase user’s understanding and awareness, which could enhance the users’ ability to make better-informed decisions.

The use of the *Training* function was reported by two articles.^35,36^ *Training* involves acquiring and practicing skills necessary for performing the desired behavior effectively. Two articles in our review specified that their apps provided *Training* through the delivery of a variety of diabetes self-management strategies and promoting learning skills. The limited use of the *Training* BCW function in T2D apps suggests a potential gap in app design, where opportunities for skills-building are not fully utilized, potentially limiting users’ ability to effectively manage their condition. *Training* was also found to be an underused intervention function in mHealth technologies within diabetes management practice, and its implementation is recommended to improve engagement.^39^

In our review, five of the nine BCW intervention functions aimed at improving users’ motivation or opportunity (ie, *Incentivization, Coercion, Restriction, Environmental Restructuring*, and *Modeling*) were not identified or reported as being used in the T2D apps. However, recent studies have highlighted the potential of *Incentivization* and *Environmental Restructuring* in other interventions, such as gestational diabetes prevention^40^ and improving adherence to antidiabetic medication.^13^ These underused BCW functions could further promote behavior change in T2D app users. For instance, *Incentivization* could be employed by offering rewards or achievements for meeting goals; *Coercion* could involve applying some notification messages for non-adherence, though this must be done with caution to avoid negative reactions. *Restriction* could be implemented by encouraging individuals to limit certain behaviors, such as unhealthy food choices. *Environmental Restructuring* could modify users’ physical or digital environments, such as adjusting app interfaces to prioritize healthier behaviors or providing contextual cues for behavior change. And *Modeling* could be used to show success stories of other users, inspiring individuals through relatable examples of healthy lifestyle adoption. Incorporating these BCW functions could further enhance the effectiveness of T2D apps in enhancing sustained positive behavior change.

Type 2 diabetes is a global concern, with estimates suggesting the number of affected individuals will nearly double in the next few decades.^1,2^ Effective mobile apps can aid in self-management, and some have already demonstrated their efficacy in improving health outcomes.^41-44^ To advance the design of these apps, it is crucial to clearly report all behavioral intervention functions, as there may be an underreporting of components in the publications and the inclusion of additional functions not explicitly detailed in the scientific literature. Incomplete or unclear descriptions of the behavioral intervention functions make it difficult to replicate studies and assess the effectiveness of diabetes apps^45^ and their various interventions’ functions. Providing too few details makes the development of evidence-based strategies difficult and limits our understanding of what truly works in behavior change for diabetes self-management. Scientists designing, developing, and testing T2D apps are encouraged to report all app functionalities, components and behavioral change functions as essential elements per the CONSORT-EHEALTH guidelines,^46^ which provide standardized reporting criteria to ensure transparency, replicability, and quality in digital health studies, as well as providing screenshots of the apps, for better clarity.

### Limitations

This literature review aimed to explore a sample of publications on T2D apps by searching three databases where research on such apps could have been published. However, the search strategy was not exhaustive as it focused only on publications with the terms “diabetes” and “app” in the title, potentially overlooking relevant studies. Despite not imposing language limitations, only one article published in a language other than English (German) was identified. Furthermore, the coding of behavior intervention functions was performed by a single researcher with a background in psychology, who categorized the explicitly reported behavior change functions based solely on the descriptions provided in the selected articles, without consulting additional information about the apps or other related publications where this information could have been reported. Thus, some behavior change functions could have been classified under more than one behavioral intervention function, introducing potential classification bias. Moreover, we did not analyze data on the reported effectiveness of these apps; future research could explore the relationship between the implementation of behavior intervention functions in T2D apps and their effectiveness.

## Conclusions

This review highlights the integration of key behavioral intervention functions crucial for supporting the self-management of type 2 diabetes. While *Enablement* and *Persuasion* are commonly used, other BCW functions such as *Incentivization, Coercion, Restriction, Environmental Restructuring*, and *Modeling* appear to be underused. The reliance on manual data entry for some of the parameters used by the app, along with incomplete reporting of behavioral intervention functions, presents challenges for assessing and replicating app effectiveness. Thus, we believe there is an unused potential for making more efficient apps in T2D and suggest that future research should explore the effects of integrating additional behavioral intervention functions.

## Figures and Tables

**Appendix C.**
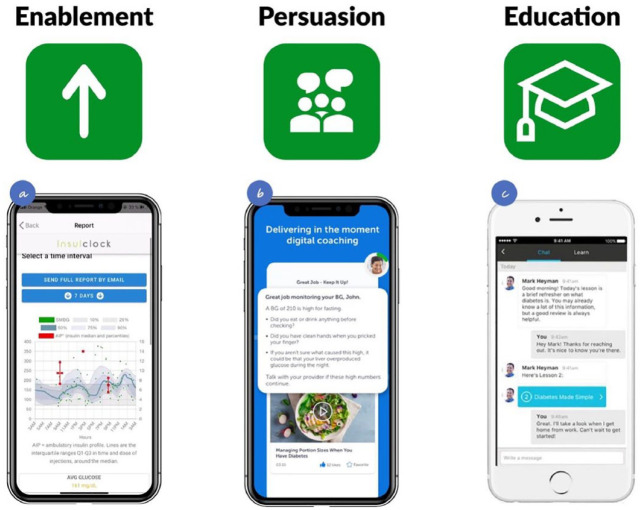
Examples of how BCW intervention functions were implemented in the apps. App: *Diabetes Control*, described in Coleone et al.^22^ Image available at: https://apps.apple.com/us/app/diabetes-control-insulclock/id1365017069. App: *WellDoc Bluestar*, described in Desveaux et al.^23^ Image available at: https://play.google.com/store/apps/details?id=com.welldoc.platform.android&hl=en_ZA&gl=US. App: *One Drop Mobile.* Image available in Kumar et al.^31^

## Appendix

**Appendix A.** Full Search Strategy.

Database	Search engine	Results
SCOPUS	TITLE (diabetes) AND TITLE (app)	388
PubMed	(diabetes[Title]) AND (app[Title])	217
PsycINFO	TI diabetes AND TI app	39
**TOTAL**		644

Search date: February 29, 2024.

**Appendix B.** Rejected Articles and Reasons for Rejection.

Article title	Exclusion reasons		
	The article is not a primary study (ie, reviews, editorials, study protocols, etc.)	The article describes an app that is not specifically for Type 2 diabetes management, or that has not been developed yet	The diabetes app does not cover all three functions (ie, management of medications, nutrition, and blood glucose control)
A cloud-based App for Early Detection of Type II diabetes with the aid of deep learning		X	
A Digital Platform and Smartphone App to Increase Physical Activity in Patients With Type 2 Diabetes: Overview Of a Technical Solution			X
A healthy lifestyle app for older adults with diabetes and hypertension: usability assessment			X
A Mobile App for Diabetes Management: Impact on Self-Efficacy Among Patients with Type 2 Diabetes at a Community Hospital			X
A Novel Diabetes Prevention Intervention Using a Mobile App: A Randomized Controlled Trial With Overweight Adults at Risk		X	
A Novel Food Record App for Dietary Assessments Among Older Adults With Type 2 Diabetes: Development and Usability Study			X
A qualitative study of users’ experiences after 3months: the first Rwandan diabetes self-management Smartphone application “Kir’App”			X
A Randomised Control Trial to Explore the Impact and Efficacy of the Healum Collaborative Care Planning Software and App on Condition Management in the Type 2 Diabetes Mellitus Population in NHS Primary Care			X
A Smartphone App to Improve Medication Adherence in Patients With Type 2 Diabetes in Asia: Feasibility Randomized Controlled Trial			X
Acceptability of an mHealth App Intervention for Persons With Type 2 Diabetes and its Associations With Initial Self-Management: Randomized Controlled Trial			X
Achieving Effective and Efficient Basal Insulin Optimal Management by Using Mobile Health Application (APP) for Type 2 Diabetes Patients in China			X
Addressing Depression Comorbid With Diabetes or Hypertension in Resource-Poor Settings: A Qualitative Study About User Perception of a Nurse-Supported Smartphone App in Peru			X
AN ANDROID APP FOR INTELLIGENT DOSAGE PLANNING IN TYPE2 DIABETES USING ANFISGA			X
App Design Features Important for Diabetes Self-management as Determined by the Self-Determination Theory on Motivation: Content Analysis of Survey Responses From Adults Requiring Insulin Therapy		X	
App-technology to increase physical activity among patients with diabetes type 2 - the DiaCert-study, a randomized controlled trial			X
Associations Between Psychosocial Needs, Carbohydrate-Counting Behavior, and App Satisfaction: A Randomized Crossover App Trial on 92 Adults With Diabetes		X	
Clinical Efficacy of a 3D Foot Scanner app for the Fitting of Therapeutic Footwear in Persons with Diabetes in Remission: A Randomized and Controlled Clinical Trial			X
Co-design of an evidence-based health education diabetes foot app to prevent serious foot complications: a feasibility study			X
Design Implications of User Experience Studies: The Case of a Diabetes Wellness App			X
Design of a Mobile App to Monitor and Control in Real Time Type 2 Diabetes Mellitus in Peru			X
Design of the Feedback Engine for a Diabetes Self-care Smartphone App			X
Designing a Mobile App to Support a Healthy Lifestyle (MAS-HeaL) for Type-2 Diabetes Patients in Malaysia			X
Development and Feasibility of an App to Decrease Risk Factors for Type 2 Diabetes in Hispanic Women With Recent Gestational Diabetes (Hola Bebé, Adiós Diabetes): Pilot Pre-Post Study			X
Development of a Small Steps for Big Changes Diabetes Prevention App: Application of the Development Phase of FASTER		X	
Diabetes App-Related Text Messages From Health Care Professionals in Conjunction With a New Wireless Glucose Meter With a Color Range Indicator Improves Glycemic Control in Patients With Type 1 and Type 2 Diabetes: Randomized Controlled Trial			X
DIABEO System Combining a Mobile App Software With and Without Telemonitoring Versus Standard Care: A Randomized Controlled Trial in Diabetes Patients Poorly Controlled with a Basal-Bolus Insulin Regimen			X
Differential influences of social support on app use for diabetes self-management – a mixed methods approach		X	
DM-calendar app as a diabetes self-management education on adult type 2 diabetes mellitus: a randomized controlled trial			X
Effect of a Smartphone App on Weight Change and Metabolic Outcomes in Asian Adults With Type 2 Diabetes A Randomized Clinical Trial			X
Effect of an app on students’ knowledge about diabetes during the COVID-19 pandemic		X	
Effect of social app-assisted education and support on glucose control in patients with coronary heart disease and diabetes mellitus			X
Effectiveness of Lilly Connected Care Program (LCCP) App-Based Diabetes Education for Patients With Type 2 Diabetes Treated With Insulin: Retrospective Real-World Study			X
Effects of Dietary App-supported Tele-counseling on Sodium Intake, Diet Quality, and Blood Pressure in Patients with Diabetes and Kidney Disease			X
Efficacy of Personalized Diabetes Self-care Using an Electronic Medical Record–Integrated Mobile App in Patients With Type 2 Diabetes: 6-Month Randomized Controlled Trial			X
Efficiency of an mHealth App and Chest-Wearable Remote Exercise Monitoring Intervention in Patients With Type 2 Diabetes: A Prospective, Multicenter Randomized Controlled Trial			X
Enhanced Self-Efficacy and Behavioral Changes Among Patients With Diabetes: Cloud-Based Mobile Health Platform and Mobile App Service			X
Enhancing type 2 diabetes treatment through digital plans of care. Patterns of access to a care-planning app over the first 3 months of a digital health intervention			X
EVALUATION OF USABILITY OF MALAYSIA DIABETES PREVENTION PROGRAM (MyDiPP) MOBILE APP – A PILOT STUDY		X	
Exploring the impact of a care planning software and app solution on the management of type 2 diabetes	X		
Feasibility and user experience of the unguided web-based self-help app ‘MyDiaMate’ aimed to prevent and reduce psychological distress and fatigue in adults with diabetes			X
General Behavioral Engagement and Changes in Clinical and Cognitive Outcomes of Patients with Type 2 Diabetes Using the Time2Focus Mobile App for Diabetes Education: Pilot Evaluation			X
Health Education for Diabetes Medication Adherence via the Whatsapp Messaging App (WEDMA) Module: A Content Validity Study	X		X
Heuristic Evaluation of a Top-Rated Diabetes Self-Management App	X	X	X
Honoring Heritage, Managing Health: A Mobile Diabetes Self-Management App for Native Americans with Cultural Sensitivity and Local Factors	X		X
Impact of a mobile app on medication adherence and adherence-related beliefs in patients with type 2 diabetes			X
Impact of My Dose Coach App Frequency of Use on Clinical Outcomes in Type 2 Diabetes			X
Implications for GP endorsement of a diabetes app with patients from culturally diverse backgrounds: a qualitative study	X	X	
Improved Glycemic Control Using a Bluetooth®- Connected Blood Glucose Meter and a Mobile Diabetes App: Real- World Evidence From Over 144000 People With Diabetes	X	X	X
Randomized, controlled trial of a digital behavioral therapeutic application to improve glycemic control in adults with type 2 diabetes.			X
Incorporating Behavioral Trigger Messages Into a Mobile Health App for Chronic Disease Management: Randomized Clinical Feasibility Trial in Diabetes			X
Incorporation of a Stress Reducing Mobile App in the Care of Patients With Type 2 Diabetes: A Prospective Study			X
Influence of Patient Characteristics and Psychological Needs on Diabetes Mobile App Usability in Adults With Type 1 or Type 2 Diabetes: Crossover Randomized Trial	X		X
Klinio mobile app for diabetes self-care: A pilot study of HbA1c improvement in type 2 diabetes patients			X
Lack of Adoption of a Mobile App to Support Patient Self-Management of Diabetes and Hypertension in a Federally Qualified Health Center: Interview Analysis of Staff and Patients in a Failed Randomized Trial	X	X	X
Managing Diabetes Using Mobiab: Long-Term Case Study of the Impact of a Mobile App on Self-management		X	
Manchester Intermittent versus Daily Diet App Study (MIDDAS): A pilot randomized controlled trial in patients with type 2 diabetes			X
Medication Adherence App for Food Pantry Clients With Diabetes: A Feasibility Study			X
Mobile App for Improved Self-Management of Type 2 Diabetes: Multicenter Pragmatic Randomized Controlled Trial			X
Mobile App for Simplifying Life With Diabetes: Technical Description and Usability Study of GlucoMan	X		
Mobile Health Monitoring: Development and Implementation of an app in a Diabetes and Hypertension Clinic	X		
Mobile phone applications and their use in the self-management of Type 2 Diabetes Mellitus: a qualitative study among app users and non-app users			X
Non Invasive Blood Glucose Detection along with an Assistive Diabetes Monitoring App	X	X	X
Novel App- and Web-Supported Diabetes Prevention Program to Promote Weight Reduction, Physical Activity, and a Healthier Lifestyle: Observation of the Clinical Application		X	X
Once-Weekly Insulin Icodec With Dosing Guide App Versus Once-Daily Basal Insulin Analogues in Insulin-Naive Type 2 Diabetes (ONWARD)			X
Optimizing diabetes wound management: Healico App and dressing materials from Urgo Medical	X		
Participatory Design of a Social Networking App to Support Type II Diabetes Self-Management in Low-Income Minority Communities	X		X
Patient Self-management of Diabetes Using the Mobile Terminal APP: a Self-controlled, Comparative Study in Fangzhuang Community Health Service Center			X
Physician-Authored Feedback in a Type 2 Diabetes Self-management App: Acceptability Study	X		X
Randomized Controlled Feasibility Study of the MyHealthAvatar-Diabetes Smartphone App for Reducing Prolonged Sitting Time in Type 2 Diabetes Mellitus			X
Real-World Evidence of a Hospital-Linked Digital Health App for the Control of Hypertension and Diabetes Mellitus in South Korea: Nationwide Multicenter Study		X	X
Real-World Evidence of Improved Glycemic Control in People with Diabetes Using a Bluetooth-Connected Blood Glucose Meter with a Mobile Diabetes Management App		X	X
Remotely Conducted App-Based Intervention for Cardiovascular Disease and Diabetes Risk Awareness and Prevention: Single-Group Feasibility Trial		X	X
Screening for mental illness using GMHAT App of patients with Type 2 diabetes mellitus at a teaching institute hospital in India – A cross sectional study			X
Self-Monitoring Diabetes-Related Foot Ulcers with the MyFootCare App: A Mixed Methods Study		X	X
Sensorimotor and Cognitive Abilities Associated With Touchscreen Tablet App Performance to Support Self-Management of Type 2 Diabetes			X
Short-Term Trajectories of Use of a Caloric-Monitoring Mobile Phone App Among Patients With Type 2 Diabetes Mellitus in a Primary Care Setting			X
Should App Self-Management Mean Self-Control? A Quantitative Study on App Supported Diabetes Self-Management		X	X
Social Support, eHealth Literacy, and mHealth Use in Older Adults With Diabetes	X	X	X
Sustained Improvements in Readings in-Range Using an Advanced Bluetooth! Connected Blood Glucose Meter and a Mobile Diabetes App: Real-World Evidence from more than 55,000 People with Diabetes	X	X	X
The ActiveAgeing Mobile App for Diabetes Self- management: First Adherence Data and Analysis of Patients’ in-App Notes		X	X
The application of social cognitive theory (SCT) to the mHealth diabetes physical activity (PA) app to control blood sugar levels of type 2 diabetes mellitus (T2DM) patients in Takalar regency			X
The effect of the smartphone app DiaCert on health related quality of life in patients with type 2 diabetes: results from a randomized controlled trial			X
The Effectiveness of an App (Insulia) in Recommending Basal Insulin Doses for French Patients With Type 2 Diabetes Mellitus: Longitudinal Observational Study			X
The Effects of a Lifestyle Intervention Supported by the InterWalk Smartphone App on Increasing Physical Activity Among Persons With Type 2 Diabetes: Parallel-Group, Randomized Trial			X
The Effects of Continuous Usage of a Diabetes Management App on Glycemic Control in Real-world Clinical Practice: Retrospective Analysis		X	
The Sukaribit Smartphone App for Better Self-Management of Type 2 Diabetes: Randomized Controlled Feasibility Study			X
Triabetes: Your Diabetes All-In-One app	X		
Usability Evaluation of Diabetes Nutriment Diary: A Mobile App for Diabetic Patients	X	X	X
Usage Patterns of GlucoNote, a Self-Management Smartphone App, Based on ResearchKit for Patients With Type 2 Diabetes and Prediabetes		X	X
Use of a Mobile Phone App to Treat Depression Comorbid With Hypertension or Diabetes: A Pilot Study in Brazil and Peru		X	X
Use of technology in prevention programs: Digital diabetes prevention - with the DIP app		X	
User Retention and Engagement With a Mobile App Intervention to Support Self-Management in Australians With Type 1 or Type 2 Diabetes (My Care Hub): Mixed Methods Study		X	X
Web versus App – compliance of patients in a telehealth diabetes management program using two different technologies		X	X
Weight loss in a digital app-based diabetes prevention program powered by artificial intelligence		X	X
